# Real-World Outcomes of an Automated Physician Support System for Genome-Driven Oncology

**DOI:** 10.1200/PO.19.00066

**Published:** 2019-07-24

**Authors:** Jessica J. Tao, Michael H. Eubank, Alison M. Schram, Nicholas Cangemi, Erika Pamer, Ezra Y. Rosen, Nikolaus Schultz, Debyani Chakravarty, John Philip, Jaclyn F. Hechtman, James J. Harding, Lillian M. Smyth, Komal L. Jhaveri, Alexander Drilon, Marc Ladanyi, David B. Solit, Ahmet Zehir, Michael F. Berger, Peter D. Stetson, Stuart M. Gardos, David M. Hyman

**Affiliations:** ^1^ Memorial Sloan Kettering Cancer Center, New York, NY; ^2^ Weill Cornell Medical College, New York, NY

## Abstract

**PURPOSE:**

Matching patients to investigational therapies requires new tools to support physician decision making. We designed and implemented Precision Insight Support Engine (PRECISE), an automated, just-in-time, clinical-grade informatics platform to identify and dynamically track patients on the basis of molecular and clinical criteria. Real-world use of this tool was analyzed to determine whether PRECISE facilitated enrollment to early-phase, genome-driven trials.

**MATERIALS AND METHODS:**

We analyzed patients who were enrolled in genome-driven, early-phase trials using PRECISE at Memorial Sloan Kettering Cancer Center between April 2014 and January 2018. Primary end point was the proportion of enrolled patients who were successfully identified using PRECISE before enrollment. Secondary end points included time from sequencing and PRECISE identification to enrollment. Reasons for a failure to identify genomically matched patients were also explored.

**RESULTS:**

Data were analyzed from 41 therapeutic trials led by 19 principal investigators. In total, 755 patients were accrued to these studies during the period that PRECISE was used. PRECISE successfully identified 327 patients (43%) before enrollment. Patients were diagnosed with 29 tumor types and harbored alterations in 43 oncogenes, most commonly *ERBB2* (21.3%), *PIK3CA* (14.1%), and *BRAF* (8.7%). Median time from sequencing to enrollment was 163 days (interquartile range, 66 to 357 days), and from PRECISE identification to enrollment 87 days (interquartile range, 37 to 180 days). Common reasons for failing to identify patients before enrollment included accrual on the basis of molecular alterations that did not match pre-established PRECISE genomic eligibility (140 [33%] of 428) and external sequencing not available for parsing (127 [30%] of 428).

**CONCLUSION:**

PRECISE identified 43% of all patients accrued to a diverse cohort of early-phase, genome-matched studies. Purpose-built informatics platforms represent a novel and potentially effective method for matching patients to molecularly selected studies.

## INTRODUCTION

Genomic data are increasingly used to guide both routine and investigational treatment decisions for patients with cancer. These therapeutic advances have been driven by a convergence of factors. Primarily, selective and potent inhibitors of a wide range of critical signaling nodes are now available in the clinic. Simultaneously, clinically validated broad next-generation sequencing platforms that permit the detection of multiple potentially actionable alterations are now accessible at the point of care.^1,2^ These improvements, however, have not been accompanied by commensurate advancements in the systems that are available to help physicians interpret and act on these data. Indeed, previous experience with patients with advanced solid tumor who undergo genome profiling suggests that only a minority—5% to 24%, depending on the breadth of sequencing and practice setting—are subsequently enrolled in genome-matched trials.^3-8^ Numerous challenges exist in identifying and enrolling patients in appropriate genome-driven clinical trials. Clinicians must correctly interpret sequencing data not only at the time of the initial results, but also longitudinally, as our understanding of genomic biomarkers and associated clinical evidence continuously evolves.^9^ Enrolling patients in trials also requires access to, and up-to-date knowledge of, a changing portfolio of studies, each with its own study-specific eligibility criteria and dynamic slot availability. Finally, all of this information must be readily accessible to the clinician at critical decision points in a patient’s care.

**CONTEXT**

**Key Objective**
New strategies to facilitate matching patients to genome-driven trials are needed to support physician decision making. Precision Insight Support Engine (PRECISE) is an automated, just-in-time, clinical-grade informatics platform that identifies and dynamically tracks patients on the basis of molecular and clinical criteria. We analyzed real-world use of this tool to determine how PRECISE facilitated enrollment in genomic-driven, early-phase trials at our center. To our knowledge, this represents the first effort to evaluate outcomes of a bioinformatics patient–trial matching platform.
**Knowledge Generated**
PRECISE identified nearly one half (43%) of all enrollment in early-phase, genome-driven studies across a wide variety of tumor types and molecular alterations, with a 5-month median time from sequencing to enrollment. A major area for improving matching efficacy is better integration of nonstructured, clinical eligibility criteria.
**Relevance**
Use of automated bioinformatics platforms represents an important means by which to increase clinical trial accrual and deliver precision oncology care to patients.

To address these challenges, multiple strategies have emerged to facilitate matching patients to genome-driven clinical trials (Appendix Table A1). The most straightforward of these approaches involves directly annotating molecular sequencing reports at initial sign-out to indicate the clinical significance of each alteration and list potentially suitable clinical trials. Multiple initiatives are underway to harmonize the annotation of individual variants and incorporate multitiered levels of evidence for actionability.^10-12^ Some commercial laboratories and academic institutions have created on-demand molecular tumor boards that review molecular sequencing reports to make treatment recommendations and manually curate existing clinical trials for alterations of interest.^13^ All of these approaches have potential limitations. Annotated molecular sequencing reports generate potentially cumbersome, long lists of interpretations, typically with no prioritization or information on study availability. These annotations are also static and can rapidly become out of date as new biologic insights emerge or novel drugs become available. Similarly, molecular tumor boards are time consuming, difficult to scale, and potentially influenced by participants’ knowledge base and anecdotal experience.

In response to these limitations and to address the ongoing unmet need of matching patients to clinical trials at our center, we created Precision Insight Support Engine (PRECISE), an automated, just-in-time, clinical-grade informatics platform to identify and dynamically track patients on the basis of molecular and clinical criteria. PRECISE was designed to empower clinical investigators to proactively identify and recruit optimal candidates to their genome-driven trials. Here, we analyze real-world use of this tool to determine how PRECISE facilitated enrollment in genome-driven, early-phase clinical trials at our center.

## MATERIALS AND METHODS

### Genomic Testing and Patient Selection

All genomic data used in patient matching were generated in the Memorial Sloan Kettering (MSK) Clinical Laboratory Improvement Amendments–certified diagnostic molecular pathology laboratory using several laboratory-developed tests reflecting the evolution of profiling technologies used during the study period. Initially, limited profiling data were generated via a mass spectrometry–based hotspot assay covering eight genes (MassARRAY; Sequenom, San Diego, CA) or an amplicon-based next-generation sequencing test covering 48 genes (TruSeq Amplicon Cancer Panel; Illumina, San Diego, CA).^14^ A customized hybrid capture-based next-generation sequencing assay (MSK-IMPACT) was later performed per previously published methods.^15^ MSK-IMPACT can identify all classes of genomic alterations—single-nucleotide variants, indels, copy number alterations, and select structural rearrangements—in up to 468 genes, depending on the assay version. Data from all next-generation sequencing assays were captured in MPath, an internally developed application that uses the open-source database management system MySQL. These data are used for clinical report sign-out and delivery to the data warehouse that PRECISE uses for patient identification.^16,17^

Sequenced patients had cancer types for which profiling was considered routine at the time performed or underwent clinical testing under a prospective institutional review board–approved protocol that was designed to evaluate the utility of profiling in these cancer types (ClinicalTrials.gov identifier: NCT01775072).

### PRECISE Platform

The PRECISE platform was available to any principal investigator of an IRB-approved therapeutic study that included molecular eligibility criteria. In situations in which PRECISE might identify patients with whom study investigators had no therapeutic relationship, an IRB-approved waiver of authorization was also required. To generate the PRECISE cohort for each study, principal investigators worked with MSK information systems to craft inclusion and exclusion criteria on the basis of molecular characteristics and other structured data elements available through the MSK data warehouse using previously published methods, listed in the Appendix.^16^

**FIG 1.**
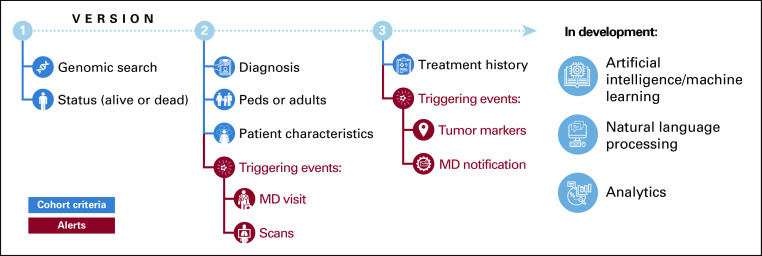
Evolution of PRECISE (Precision Insight Support Engine) functionality. Throughout the development of PRECISE, multiple functionalities were gradually enhanced. The initial iteration (version 1) of PRECISE involved generating cohorts on the basis of complex genetic and clinical criteria defined by the study’s principal investigator (PI), which could then be sent to the PI at defined intervals. The capability of PRECISE was later enhanced (version 2) to enable PI notifications triggered by certain events of interest, such as an upcoming patient appointment or computed tomography scan. Present day (version 3) PRECISE can also incorporate a patient’s prior treatment history and allows for direct notification of the patient’s treating oncologist that a patient may be eligible for a study, often prompting an exchange between the treating oncologist and PI that initiates the patient’s future enrollment. Future development of PRECISE includes harnessing machine learning algorithms and continuous feedback loop analytics to enhance efficiency and accuracy of trial–patient matches. MD, medical doctor; Peds, pediatrics.

The functionality of the PRECISE platform evolved during the study period on the basis of user feedback (Fig 1). In total, PRECISE underwent three major functionality releases. Initially, in 2012, PRECISE could generate a list of all molecularly eligible patients who were alive at the time of the initial cohort creation. Investigators could opt to receive notifications of newly identified patients at a self-chosen frequency, typically between daily and weekly. Beginning in 2014, PRECISE was enhanced to offer notifications, customized per study, of potentially clinically important events that could prompt a treatment change, such as upcoming appointments or restaging scans. For most of the study duration, PRECISE notifications were provided directly to the research team, which empowered them to further screen potential patients for appropriateness and notify treating physicians accordingly. Beginning in June 2016, PRECISE was again enhanced to permit individualized, patient-specific, customized notifications to be sent directly to the primary treating oncologist. Notifications could be automatically triggered by multiple prespecified events, including sign-out of new sequencing data that identify a qualifying alteration or consecutively rising tumor markers. Use of this direct-to-primary oncologist notification function was at the discretion of the research team. All notifications to the research team and the primary oncologist were generated as emails and did not directly integrate with the electronic medical record.

### Cohort Eligibility, Data Collection, and Statistical Analysis

All PRECISE cohorts for early-phase studies—pilot, phase I, and phase I and II—that included at least one genomic eligibility criterion and that were created after April 16, 2014, were included for analysis. The data lock was performed on January 31, 2018. Therapeutic protocol documents were manually reviewed to ensure that the studies met these criteria.

Data on PRECISE cohort criteria, use, and notification were parsed from usage logs. Patient enrollment data were obtained from the MSK clinical research database, which centrally maintains patient registrations to all therapeutic studies at MSK, including the date of enrollment. An internally developed MPath application was used to obtain the date that sequencing data was signed out and entered into the medical record for each patient, as well as the specific qualifying genomic alteration present.

The MSK institutional review board evaluated and approved a retrospective research protocol to evaluate PRECISE platform outcomes. Primary outcome measure was to determine what proportion of patients who were enrolled in the evaluated genome-driven studies was facilitated by PRECISE. For the purpose of this analysis, enrollment was considered to be facilitated if PRECISE identified the patient as eligible and generated a notification to either the research team or the primary oncologist before the enrollment date. Studies with no accrual during the evaluation period were excluded. Secondary outcome measures included determining the time from sequencing and PRECISE identification to patient enrollment in the relevant therapeutic study. Timing was expressed using descriptive statistics. To identify areas for future PRECISE functionality enhancement, reasons for the failure of PRECISE identify eligible patients before study enrollment were also explored through manual record review.

## RESULTS

### Study Characteristics

A total of 41 therapeutic trials used PRECISE for genomic matching during the study period (Table 1). Approximately one half (22 [54%] of 41) of studies were phase I, and 61% (25 of 41) included multiple tumor types. Of disease-specific studies, the most common tumor types included breast cancer (five [12%] of 41) and non–small-cell lung cancer (four [10%] of 41). Most studies (35 [85%] of 41) evaluated small-molecule monotherapy, and an additional 9.8% (four of 41) included a small-molecule monotherapy in combination with other investigational agents. The first trial opened to accrual on July 13, 2012, and the last began accruing on October 11, 2017. Of the 41 trials, 14 used PRECISE from initial study opening and the rest of the trials began using the system after protocol activation. Eight of 41 trials used the direct-to-oncologist notification system.

**TABLE 1.**
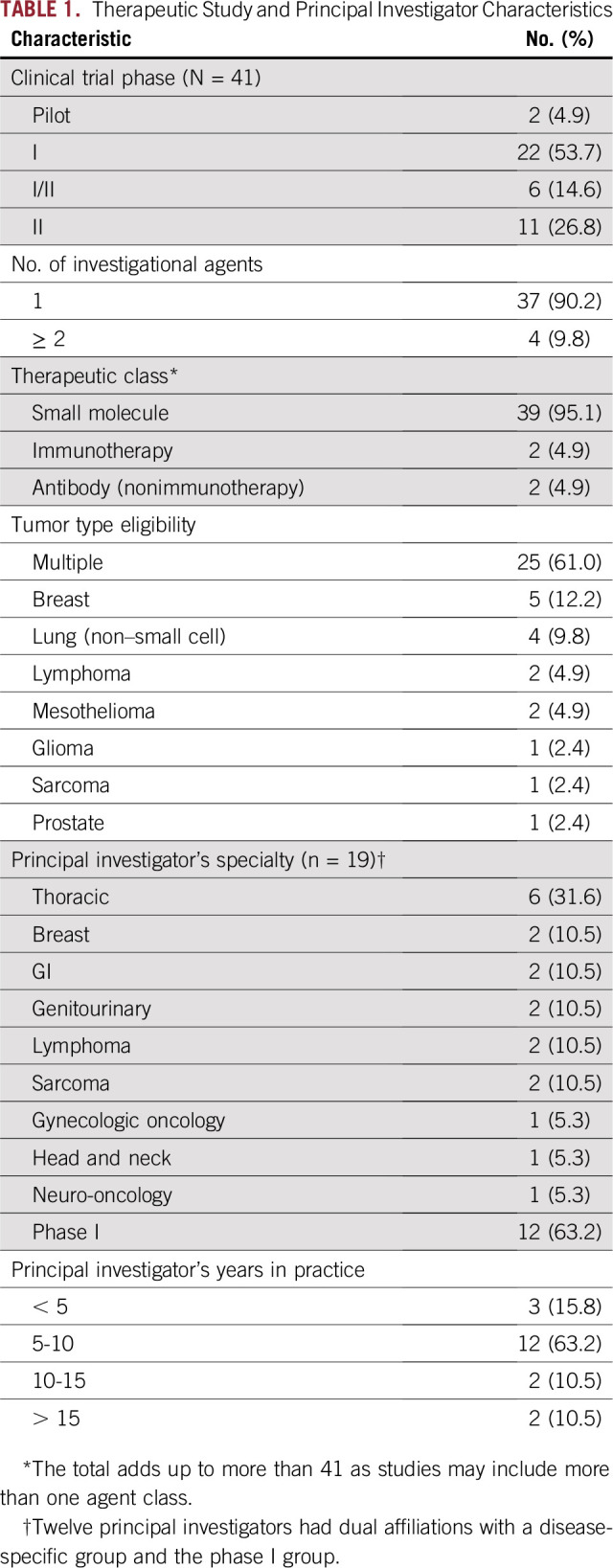
Therapeutic Study and Principal Investigator Characteristics

### Principal Investigator Characteristics

The 41 therapeutic trials were led by 19 unique principal investigators (PIs) who individually led between one and five studies (Table 1). Disease specialties that were represented included thoracic (31.6%), breast (10.5%), GI (10.5%), genitourinary (10.5%), lymphoma (10.5%), sarcoma (10.5%), gynecologic (5.3%), head and neck (5.3%), and neuro-oncology (5.3%). Nearly two thirds of PIs (12 [63.2%] of 19) were also investigators in the early drug development (phase I) service. Median time since the completion of terminal subspecialty training for PIs was 7 years (range, 2 to 27 years).

### Patient Matching

**FIG 2.**
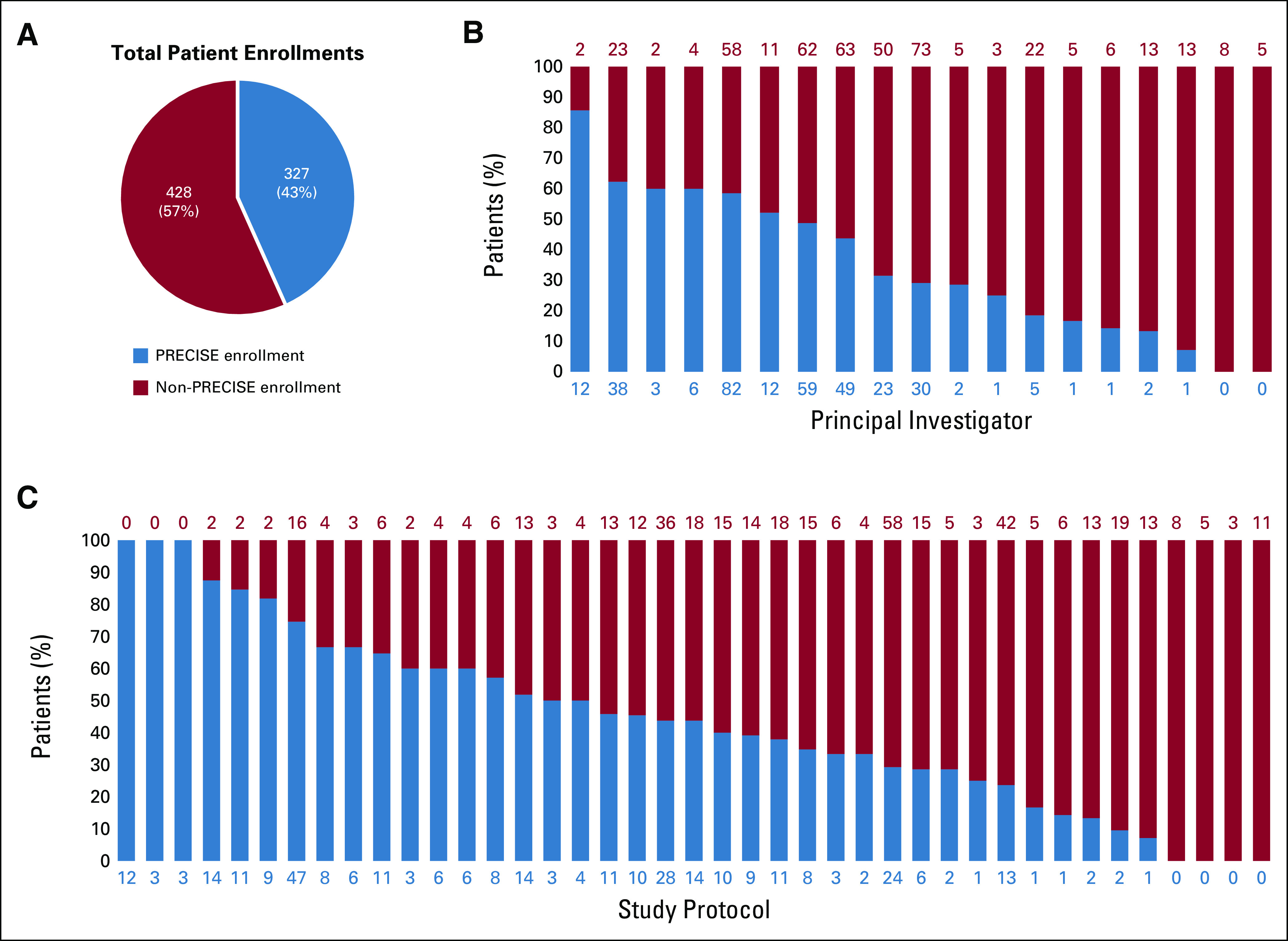
Source of patient enrollment by genome-driven study. (A) In aggregate, 43% (327 of 755) of all patient enrollments were facilitated by Precision Insight Support Engine (PRECISE). (B) Each column depicts patient enrollments by study principal investigator, with patient enrollment facilitated by PRECISE shaded in blue and patient enrollment not facilitated by PRECISE shaded in red. The absolute number of patients in each category is labeled above (non-PRECISE) and below (PRECISE enrollment) each column. (C) Each column represents a unique study, with the absolute number of patients in each category labeled above (non-PRECISE) and below (PRECISE enrollment) each column.

During the study period, a total of 755 patients who were treated primarily by 150 unique oncologists were enrolled in 41 trials. PRECISE prospectively identified 43% (327 of 755) of cases before patient enrollment, successfully notifying study investigators and/or the primary oncologist of the potential match (Fig 2). These patients had a wide range of tumor types and harbored multiple classes of genomic alterations that targeted a variety of genes (Table 2). Reflecting eligibility criteria among the trials, breast cancer (76 [23%] of 327) and lung cancer (55 [17%] of 327) were the most common tumor types among enrolled patients identified by PRECISE. Accounting for patients with more than one eligible molecular alteration, ERBB2 (71 [21%] of 335) was the most common genomic alteration, followed by PIK3CA (47 [14%] of 335).

**TABLE 2.**
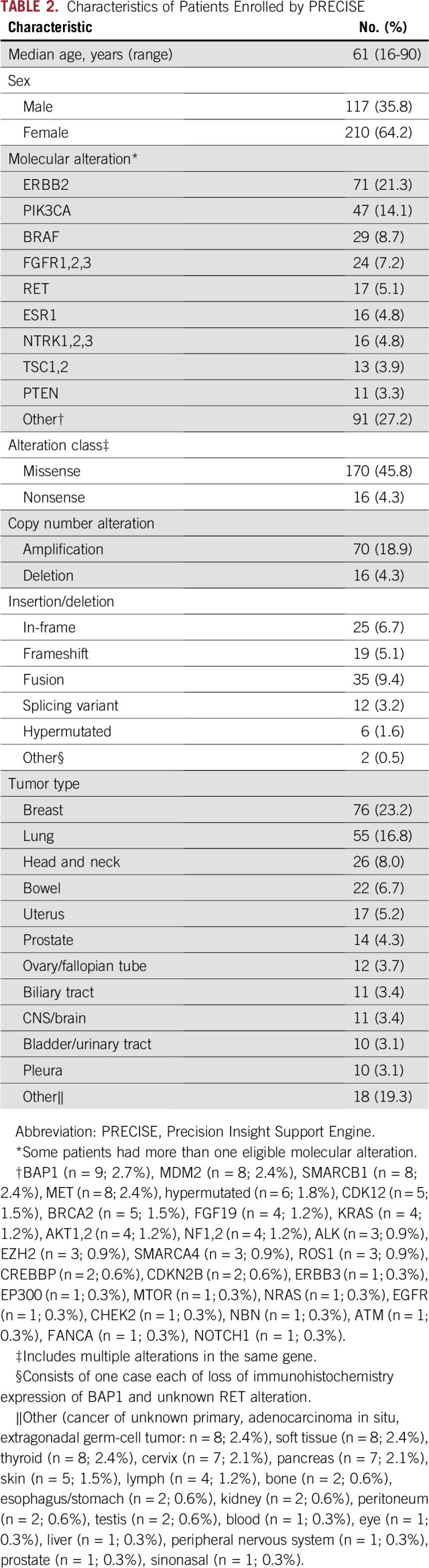
Characteristics of Patients Enrolled by PRECISE

At the individual protocol level, the percent of patients identified by PRECISE before enrollment ranged from 0% to 100%. Multiple reasons for nonidentification by PRECISE existed. Patients in whom the genomic alteration that ultimately led to enrollment did not meet the pre-established PRECISE molecular criteria, as defined by the investigator, accounted for 33% (140 of 428) of missed cases. A lack of internal sequencing at the time of accrual accounted for 30% (127 of 428) of missed cases, predominantly among patients who enrolled on the basis of genomic profiling that was performed outside the institution and was therefore not available for parsing by PRECISE. Another 23% (100 of 428) of cases was missed because PRECISE cohort criteria that would have included the patient were amended only after the time of patient enrollment. Several other technical and clinical reasons accounted for the remaining 14% (61 of 428) of cases, the majority of which (50 of 61) consisted of patients who did not meet clinical criteria available for capture by PRECISE, such as presence of triple-negative breast cancer, which was not always readily documented in the medical record.

**FIG 3.**
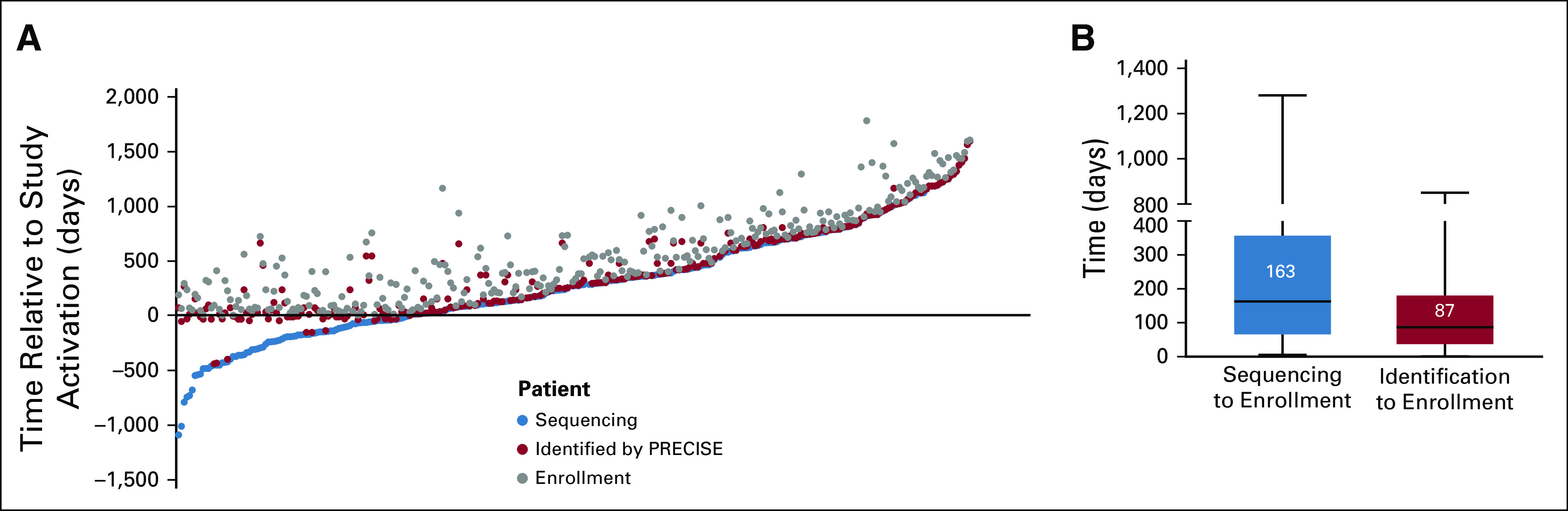
Time courses to patient enrollment. (A) Scatterplot depicting the timing of enrollment by individual patient relative to study activation (opening for accrual). Each series of three dots on a single vertical axis represents a single patient’s course, from initial sequencing (blue dot) to identification by PRECISE (Precision Insight Support Engine; red dot) to enrollment in the study (gray dot). (B) Box and whisker plot showing the interval of time from sequencing to enrollment in the study (left; median, 163 days) and from identification by PRECISE to enrollment in the study (right; median, 87 days).

To further understand how physicians and patients use tumor genomic data to guide investigational treatment decisions, we evaluated the timing of enrollment relative to two important milestones: the completion of sequencing and the initial PRECISE identification. Upon evaluation of the 327 patients who were identified by PRECISE and successfully enrolled in a genome-driven study, there was significant variability in time intervals between these three events at the individual patient level. Median time from sequencing to therapeutic enrollment was 163 days (interquartile range, 66 to 357 days; range, 5 to 1,281 days) and from PRECISE identification to enrollment 87 days (interquartile range, 37 to 180 days; range, 1 to 850 days; Fig 3). Reasons for delay from sequencing and PRECISE identification to study enrollment included the availability of alternative routine therapy or a lack of need for treatment among patients without evidence of active disease.

### Causes of Patient Attrition Before Enrollment

**FIG 4.**
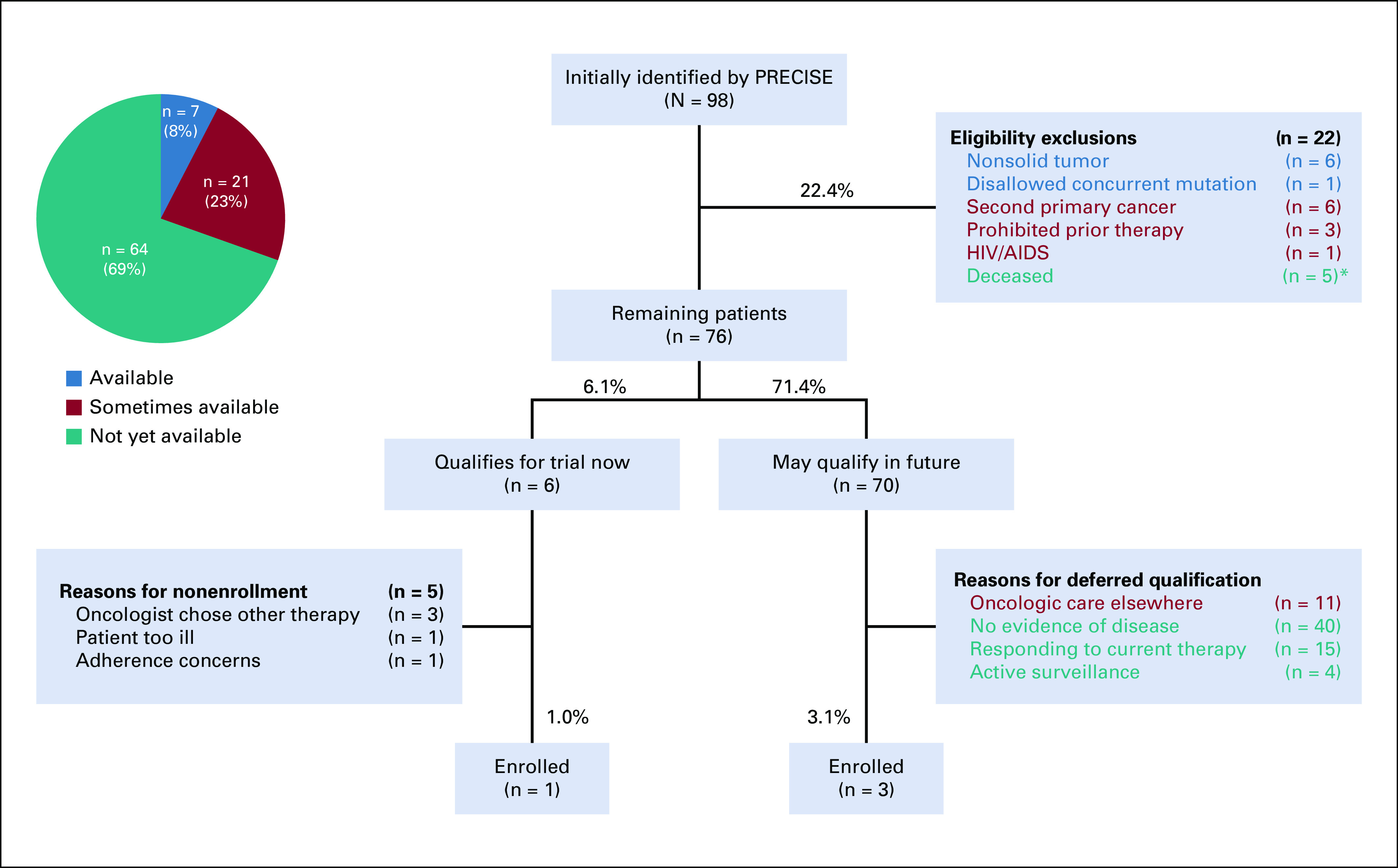
CONSORT diagram. Understanding factors that contribute to cohort patient attrition. Diagram depicts factors that led to attrition from a representative Precision Insight Support Engine (PRECISE) cohort for a phase I and II study. Reasons for permanent or temporary exclusion from study qualification are depicted in blue (criteria that is available for use by PRECISE), red (criteria that is sometimes available and could potentially be captured as an extractible structured data element), or teal (criteria that is not yet available for use by PRECISE). (*) Listed as alive in electronic medical record but actually deceased.

To better understand the reasons why patients who were identified by PRECISE did not subsequently enroll in the relevant therapeutic study, a representative cohort involving a multitumor phase I and II expansion study of a targeted small molecule was selected for manual record review. In total, PRECISE initially identified 98 patients on the basis of the genomic inclusion criteria and being listed as alive (Fig 4). Of these 98 patients, 22 were immediately determined to be permanently ineligible or excluded as a result of a static characteristic that rendered the patient ineligible for the trial indefinitely. These included having a second primary cancer (n = 6), a nonqualifying malignancy (n = 6), being deceased but not listed as such in the medical record (n = 5), and other reasons (n = 5). Reasons for permanent exclusion in 32% (seven of 22) of patients involved structured data elements that were readily available for use by PRECISE—nonsolid tumor and disallowed concurrent mutation—but that were not included in the initial PRECISE cohort criteria. Another 45% (10 of 22) involved a data element that was sometimes available—second primary cancer, prohibited prior therapy, and HIV/AIDS—for use but not under all circumstances. For example, exclusions on the basis of prior therapy will miss agents administered at other medical centers or some oral anticancer agents, especially if dispensed by third-party pharmacies.

After permanent exclusions, 76 potentially eligible patients remained. Upon manual review, 78% (59 of 76) of patients did not immediately qualify for treatment at the time of the initial PRECISE identification on the basis of not requiring active therapy (n = 44) or ongoing response to current therapy (n = 15). An additional 14% (11 of 76) were lost to follow-up. Collectively, 84% (59 of 70) of these temporary exclusion criteria are not consistently available for use by PRECISE, primarily because the current disease status of the patient is not captured as a structured data element in the medical record. Of the six patients who met immediate eligibility criteria and needed new treatment, only one enrolled, with the remainder either electing alternative therapies, rapidly deteriorating, or deemed inappropriate because of other issues, such as prior nonadherence. In total, only 4% (four of 98) of patients who were initially identified by PRECISE successfully enrolled in this study.

## DISCUSSION

**FIG A1.**
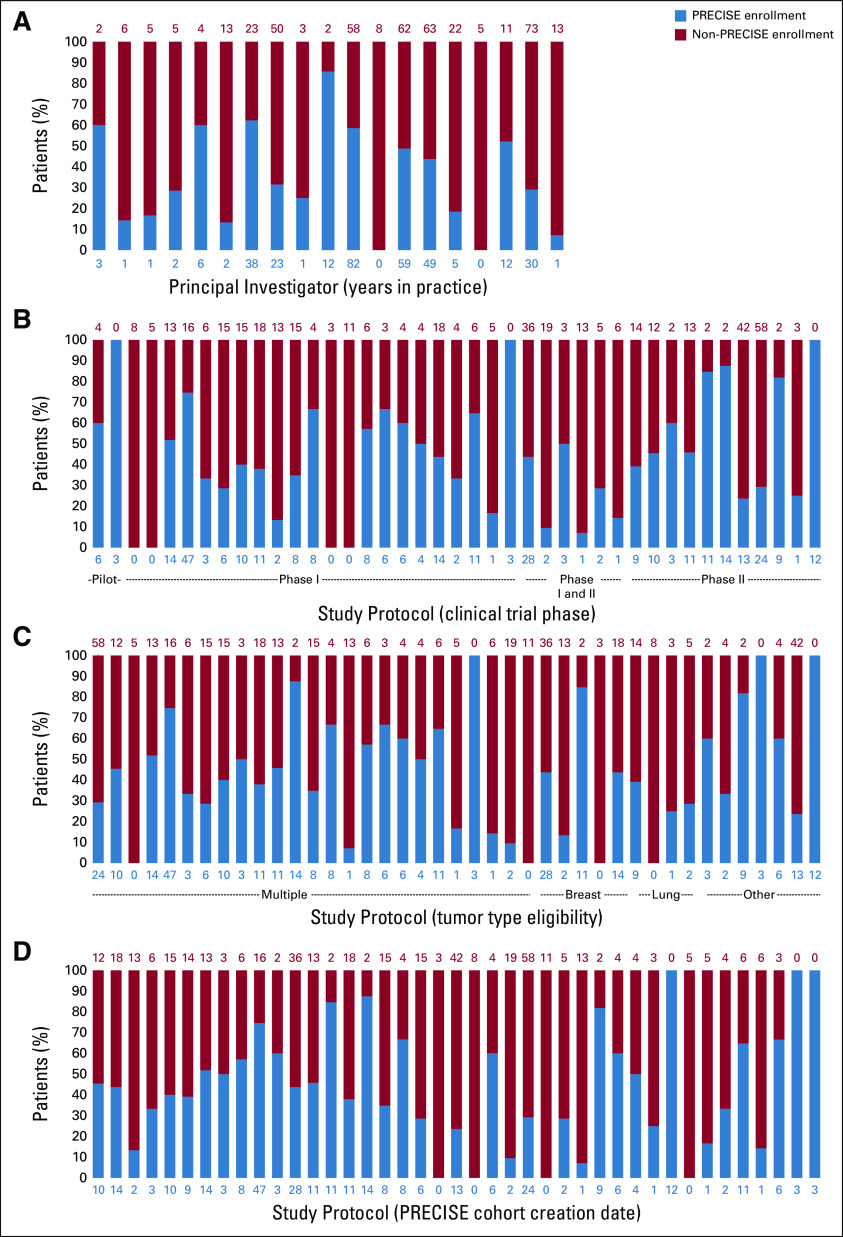
Source of patient enrollment by principal investigator (PI), study characteristic, and PRECISE (Precision Insight Support Engine) cohort creation date. (A) Each column depicts patient enrollments by study PI, with PIs with the most years in practice on the left and the fewest years in practice on the right. Patient enrollment facilitated by PRECISE is shaded in blue and patient enrollment not facilitated by PRECISE is shaded in red. The absolute number of patients in each category is labeled above (non-PRECISE) and below (PRECISE) enrollment each column. (B-D) Each column represents a unique study, with the type of study (pilot, phase I, phase I and II, or phase II; [B]), tumor type eligibility (multiple, breast, lung, or other; [C]), and date of cohort creation (earliest on the left; [D]). The earliest cohort was created on April 16, 2014, and the latest cohort was created on October 11, 2017. The absolute number of patients in each category is labeled above (non-PRECISE) and below (PRECISE enrollment) each column.

Analyzing accrual patterns to early-phase, genome-driven studies using PRECISE during a 4-year period, we found that PRECISE helped facilitate nearly one half (43%) of all enrollment in these studies (Appendix Fig A1). To our knowledge, this report represents the first effort to evaluate real-world outcomes of an automated, just-in-time, clinical-grade informatics platform to facilitate patient matching. We also discovered that a significant time interval often elapses between the initial generation of tumor genomic data and subsequent enrollment in a matched study. Specifically, among those who ultimately accrue to a matched study, median duration from data generation to enrollment was 5 months, with some outliers enrolling up to 42 months later. These data emphasize the importance of supporting physician decision making longitudinally through a patient’s entire treatment.

Although these pilot data are encouraging, our analysis also identified areas of ongoing challenge for such matching systems as PRECISE. Through manual curation of one representative PRECISE cohort, we found that only 4% of patients who were initially identified as potentially eligible ultimately enrolled. Of importance, nearly three quarters of initially identified patients were not immediately eligible on the basis of clinical factors that were not readily available for use by PRECISE, most commonly related to challenges in algorithmically defining patient disease status. The result is that PRECISE had a high false-positive rate that may ultimately limit the utility of this system for some indications, particularly for recruiting patients with more prevalent genomic alterations. Indeed, a previous analysis of overall match rates at our institution that was conducted during roughly the same time period found a match rate of only 11%, despite 37% of patients harboring a potentially actionable alteration.^5^ Taken together, these data demonstrate that even with the use of a sophisticated decision support system, there is an ongoing need for additional improvement in the methodologies to match patients to clinical trials on the basis of tumor genomic and clinical information. A major area necessary for improving matching efficiency by automated informatics platforms is better integration of additional clinical eligibility criteria, such as disease status and response to therapy. This requires developing agreed-upon standards, including discrete, structured criteria, for extracting clinical data from the electronic medical record. In the future, leveraging natural language processing and information extraction technologies may also enhance the ability to accurately capture unstructured data.

This analysis has some important limitations. Foremost, we considered any patient about whom PRECISE successfully notified the PI or treating physician of potential eligibility before enrollment as an accrual that was potentially facilitated by the system. However, we cannot determine exactly how many of these enrollments might have occurred without the use of this system. Indeed, our study was retrospective in design and we cannot definitively make conclusions on the incremental value of PRECISE. Moreover, this analysis represents real-world use of PRECISE by each PI, who were responsible for setting his or her own cohort criteria and using the results as he or she felt best complimented the practice. Finally, PRECISE was not one system but rather an evolving platform during the study duration. Additional evaluation is needed to determine how the utility of this system is affected by recent features, such as full automation of direct-to-treating physician alerts triggered by critical events, like scans that show progression or rising tumor markers.

PRECISE is not the only system designed to address the emerging need of matching patients to relevant clinical trials on the basis of the patient’s genomic profile. Several other centers have developed strategies by which to achieve this goal that range from on-site or virtual molecular tumor boards to automated matching platforms^13,18-23^ (Appendix Table A1). Each of these systems offers physician decision support that aims to bridge the gap between genomic alteration detection and identification of the appropriate genome-driven therapy. Potential advantages of an automated informatics approach include scalability and the ability to track patients longitudinally and respond to changing molecular and clinical data.

In summary, this pilot study of real-world use of PRECISE to guide enrollment in genome-driven studies suggests that this type of real-time decision support system can meaningfully facilitate patient enrollment. As the use of genomic profiling in oncology care increases, new tools are necessary to maximize the utility of this information and bring precision oncology care to patients. This study reinforces the potential of automated bioinformatics platforms as an important means to increase clinical trial accrual and enhance the delivery of precision oncology care to patients.

## Figures and Tables

**TABLE A1.**
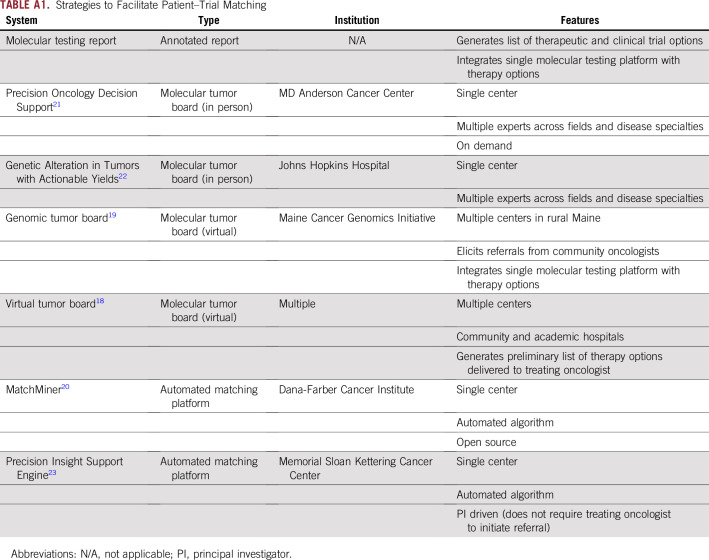
Strategies to Facilitate Patient–Trial Matching

## APPENDIX

### PRECISE Technical Specifications and Workflow

PRECISE (Precision Insight Support Engine) leverages an existing IBM DB2 data warehouse and was designed using open-source software where possible. Notifications are generated from scheduled queries and use an open-source Java e-mail package implemented as a database user-defined function to send emails from the SQL statement. A Web application using open-source JavaScript libraries allows system administrators to capture cohort logic and settings and provides end users the ability to annotate patient status for their study. Additional details on the technical specifications of PRECISE, workflow support, and the notification system are described in previously published methods.^16^
